# Feasibility of trial procedures for a randomised controlled trial of a community based group exercise intervention for falls prevention for visually impaired older people: the VIOLET study

**DOI:** 10.1186/s12877-018-0998-6

**Published:** 2018-12-12

**Authors:** Nicola Adams, Dawn A. Skelton, Denise Howel, Cathy Bailey, Rosy Lampitt, Tony Fouweather, Joanne Gray, Dorothy Coe, Jennifer Wilkinson, Sheena Gawler, Lex D. de Jong, Heather Waterman, Vincent Deary, Michael Clarke, Steve W Parry

**Affiliations:** 1 Faculty of Health and Life Sciences, Northumbria University, Newcastle upon Tyne, NE7 7XA UK; 2 Institute of Applied Health Research, School of Health & Life Sciences, Glasgow Caledonian University, Glasgow, UK; 3 Institute of Health and Society, Baddiley-Clark Building, Newcastle University, Newcastle upon Tyne, UK; 4 Newcastle Clinical Trials Unit, Newcastle University, 1-4 Claremont Terrace, Newcastle upon Tyne, UK; 5 School of Physiotherapy and Exercise Science, Curtin University, Bentley, Western Australia; 6 Healthcare Sciences, Cardiff University, Cardiff, UK; 7 Newcastle Hospitals NHS Foundation Trust, Newcastle upon Tyne, UK; 8 Institute for Ageing and Health, Newcastle University, Newcastle upon Tyne, UK

**Keywords:** Falls management, Exercise, Visual impairment, Older people, Feasibility clinical trial

## Abstract

**Background:**

Visually impaired older people (VIOP) have a higher risk of falling than their sighted peers, and are likely to avoid physical activity. The aim was to adapt the existing Falls Management Exercise (FaME) programme for VIOP, delivered in the community, and to investigate the feasibility of conducting a definitive randomised controlled trial (RCT) of this adapted intervention.

**Methods:**

Two-centre randomised mixed methods pilot trial and economic evaluation of the adapted group-based FaME programme for VIOP versus usual care. A one hour exercise programme ran weekly over 12 weeks at the study sites (Newcastle and Glasgow), delivered by third sector (voluntary and community) organisations. Participants were advised to exercise at home for an additional two hours over the week. Those randomised to the usual activities group received no intervention.

Outcome measures were completed at baseline, 12 and 24 weeks. The potential primary outcome was the Short Form Falls Efficacy Scale – International (SFES-I).

Participants’ adherence was assessed by reviewing attendance records and self-reported compliance to the home exercises. Adherence with the course content (fidelity) by instructors was assessed by a researcher. Adverse events were collected in a weekly phone call.

**Results:**

Eighteen participants, drawn from community-living VIOP were screened; 68 met the inclusion criteria; 64 participants were randomised with 33 allocated to the intervention and 31 to the usual activities arm.

94% of participants provided data at the 12 week visit and 92% at 24 weeks. Adherence was high. The intervention was found to be safe with 76% attending nine or more classes. Median time for home exercise was 50 min per week.

There was little or no evidence that fear of falling, balance and falls risk, physical activity, emotional, attitudinal or quality of life outcomes differed between trial arms at follow-up.

**Conclusions:**

The intervention, FaME, was implemented successfully for VIOP and all progression criteria for a main trial were met. The lack of difference between groups on fear of falling was unsurprising given it was a pilot study but there may have been other contributory factors including suboptimal exercise dose and apparent low risk of falls in participants. These issues need addressing for a future trial.

**Trial registration:**

Current Controlled Trials ISRCTN ID: 16949845 Registered: 21 May 2015.

## Background

Falls in older people are common [1, 2] and are associated with considerable morbidity and mortality [3], with approximately 10% of falls resulting in fractures [4]. Costs account for 0.85–1.5% Western economies’ total health-care expenditure [5]. In the UK, in 2015, falls in the over 65 s were estimated to cost £4.6 million a day [6] and thus represent a high cost to health and social care budgets [7, 8].

The incidence and prevalence of older people with significant sight loss is increasing [9]. Older people with visual impairment have a 1.7 times higher risk of falling than the general population, requiring more hospital and nursing home admissions. More contact with their general practitioner (GP) than sighted peers is also reported. [10]. Eight per cent of falls-related hospital admissions are likely to occur in people who are visually impaired [8], which accounts for approximately 21% of the total cost of treating falls. Visual impairment thus acts as an independent risk factor for falls [11–13] with falls risk factors including poor visual acuity and contrast sensitivity, decreased depth perception and reduced visual field [14–18], in addition to more general factors such as muscle weakness and balance.

Fear of falling (FoF), an umbrella term for the psychosocial consequences of falls, is common and a significant predictor of a future fall alongside a cycle of restricting daily activity and mobility with loss of confidence, reduced social participation, increased frailty and reduced quality of life [19–22]. A vision charity found that older people are highly likely to avoid activity because of their visual impairment [23]. Anxiety and depression are also common in those with visual impairment with concomitant reduced activity [3].

A Cochrane review found that exercise can reduce fear of falling in the short term, with insufficient evidence for longer term efficacy [24, 25]. A high risk of bias was noted in most included trials. There are also limited health economic data about fear of falling interventions [26, 27]. Multifactorial falls intervention programmes are effective in reducing falls among older people [28–31]. A Cochrane systematic review reported that home safety assessment and multi-component group and home-based exercise programmes reduce the rate of falls and the risk of falling in community dwelling older people [30]. For example, a New Zealand based randomised controlled trial (RCT), [32], showed no benefit from a multicomponent home-based exercise programme in visually impaired participants, though those with stricter adherence to the exercise programme had fewer falls. Adherence to the home based exercise programme in VIOP was poor with only 18% of VIOP completing all home exercise sessions over a year period. In a subsequent 3-armed UK based feasibility trial (VIP2 UK), all participants who completed the trial reported partially or completely adhering to home safety recommendations, but evidence for adherence to home exercise was equivocal [33].

Older people with visual impairment are therefore at higher risk of falls and fear of falling and its associated adverse psychosocial effects. Evidence of the effectiveness of exercise programmes and adherence to them in such individuals is lacking, though there is some evidence to suggest that group based exercise may be of more benefit than individual programmes. In addition, there is an issue with case ascertainment in those with falls and visual impairment, with only half of falls clinics in the UK assessing routinely for visual impairment [34].

The current study used a known effective community-based exercise intervention, routinely used in falls services in the UK [35]. The aims of the study were to conduct a feasibility study to inform the design and conduct of a future definitive multicentre randomised controlled trial (RCT) and economic evaluation of an adapted group-based exercise programme to prevent falls and reduce fear of falling among VIOP. Specific aims of the feasibility study were to assess recruitment and retention to the study, willingness to be randomised and to test the trial methodology, including the identification of candidate outcome measures and rehearse the methodology for cost effectiveness analysis. Participants’ adherence to the exercise programme was also examined. The acceptability of the intervention was examined qualitatively, and will be reported in a separate paper.

## Methods

### Study design

The study adheres to CONSORT guidelines for the design and reporting of clinical trials.

This study adapted an existing effective group-based health promotion intervention (FaME) for VIOP. Because of the lack of relevant information for a full RCT on this topic, a randomised mixed methods feasibility study was designed to inform the design and conduct of a future definitive multi-centre RCT on an adapted version of a community-based exercise intervention for falls management, the FaME programme.

The design was a two-centre (Newcastle and Glasgow) randomised pilot trial and economic evaluation of an adapted exercise programme for older visually impaired people versus no intervention with embedded qualitative evaluation. Interviews were conducted to explore acceptability and applicability of the intervention, the research methods and the outcome measures. The results of these interviews are being reported in a separate paper.

Visually impaired community-dwelling older people were recruited from the 2 study sites and were randomised into one of two groups. Group One was a 12-week exercise programme (one-hour session per week), and Group Two was usual activities.

### Inclusion and exclusion criteria

The inclusion and exclusion criteria have been published in a previous protocol paper [36]. In brief these were: aged 60 years and over, community dwelling and attending a low vision clinic and/or were members of organisations for the visually impaired. Exclusion criteria were acute or uncontrolled medical conditions and inability to comprehend simple movement instructions.

### Ethical approval and consent to participate

Favourable ethical opinion from the Newcastle and North Tyneside Research Ethics Committee and R&D approval was obtained prior to commencement of the intervention. Glasgow Caledonian University was approved as a non-NHS site with local ethics approval.

Information sheets were provided to all eligible participants and written informed consent obtained prior to any study procedures. Signed or verbal consent was sought and if participants were unable to sign, consent was sought by a third party who signed the witness section of the consent form.

### Identification, screening, and recruitment

Previous studies have identified that recruitment and adherence in frail older people can be difficult, although data for recruitment and retention rates in VIOP was relatively unknown. Within the VIP2UK study [33], only 10% of those initially screened and 51% of eligible participants agreed to take part. Recruitment in the current study was from both National Health Service (NHS) and non NHS sources, in order to maximise recruitment as this has been found to be difficult in previous trials.

The Ophthalmology Department in Newcastle, Newcastle Society for Blind People (NSBP), and Visibility in Glasgow identified potential participants and passed on the expressions of interest to the research team. VIOP who expressed an interest in participating in the study received further detailed information (in English only).

Following initial low referrals from the Newcastle Low Vision Clinic, and with appropriate ethical approval, Eye Clinic Liaison Officers (ECLOs) were empowered to identify and approach potential participants. With permission, expressions of interest were forwarded to the research team who assessed for eligibility in the same way as the participants identified through the low vision clinic and NSBP.

Participants gave permission for the study team to contact their General Practitioner (GP), via letter, to ensure medical fitness of the participant to take part in the study, and once eligibility confirmed, the participant was randomised.

### Randomisation

Randomisation has been previously described [36], was stratified by centre and was administered centrally via Newcastle Clinical Trials Unit using a secure web based system using a blocked allocation system to allocate participants to the two groups. Participants were informed of their allocated treatment group following randomisation.

### Study intervention

The logistical delivery of the group-based Falls Management Exercise (FaME) programme was adapted for VIOP by stakeholders and is reported in a separate paper. The exercise component and progression content of FaME remained as the original. The exercise programme (the intervention) consisted of one hour weekly sessions over 12 weeks and these were held in community venues with a maximum capacity of ten participants per group. Two exercise groups were held at each site, with a third in Newcastle added to maximise recruitment. Participants were offered taxi transport and also brought a companion or support if they wished.

All participants completed a health screening tool, normally administered by the exercise instructors prior to delivery of the FaME programme. The exercises consisted of balance specific, individually-tailored and targeted training for dynamic balance, strength, endurance, flexibility, gait and functional skills, training to improve ‘righting’ or ‘correcting’ skills to avoid a fall and backward-chaining i.e. retraining of the ability to get down to and up from the floor. Functional floor exercises and adapted Tai Chi exercises were also carried out with progressively more challenging content over the 12 weeks. Resistance bands and mats were used [37].

Participants were also advised to exercise at home for up to two hours per week. The exercises were to be performed if possible daily, on the days the participant was not attending the exercise class. All home programmes contained ‘prompts’ that linked exercises to daily tasks e.g. performing heel raises whilst waiting for the kettle to boil, in order to improve adherence. Exercises were provided in a large text booklet, DVD or audio format. Exercises were designed to be completed in 10 to 20 min blocks, becoming more challenging and graduating into longer periods. Thus the intervention comprised up to 36 h over the 12 week period (with full adherence), concordant with current evidence [38].

### Control group

Those participants who were randomised to the usual activities group received no intervention and continued with their usual activities. They were offered an equivalent exercise programme after the 24-week follow-up data collection at both sites.

### Outcomes

The progression criteria to judge the feasibility of progressing to a full trial were that following the six-month follow-up data collection [36]:

1. ≥50% of eligible participants recruited into the feasibility study;
2. ≥70% of the participants in the intervention arm completed nine to twelve sessions in the exercise programme.
3. ≥70% of participants had data collected on main outcomes at six-month follow-up;
4. < 10% of serious adverse events deemed due to the intervention.

### Outcome measures

Following consent, a researcher assessed all participants, either on site or in their own home, depending on the participants’ preferences. Information on demographics, co-morbidities, current medication, socio-economic information and the main study outcome measures were collected at the baseline visit.

**Fig. 1:**
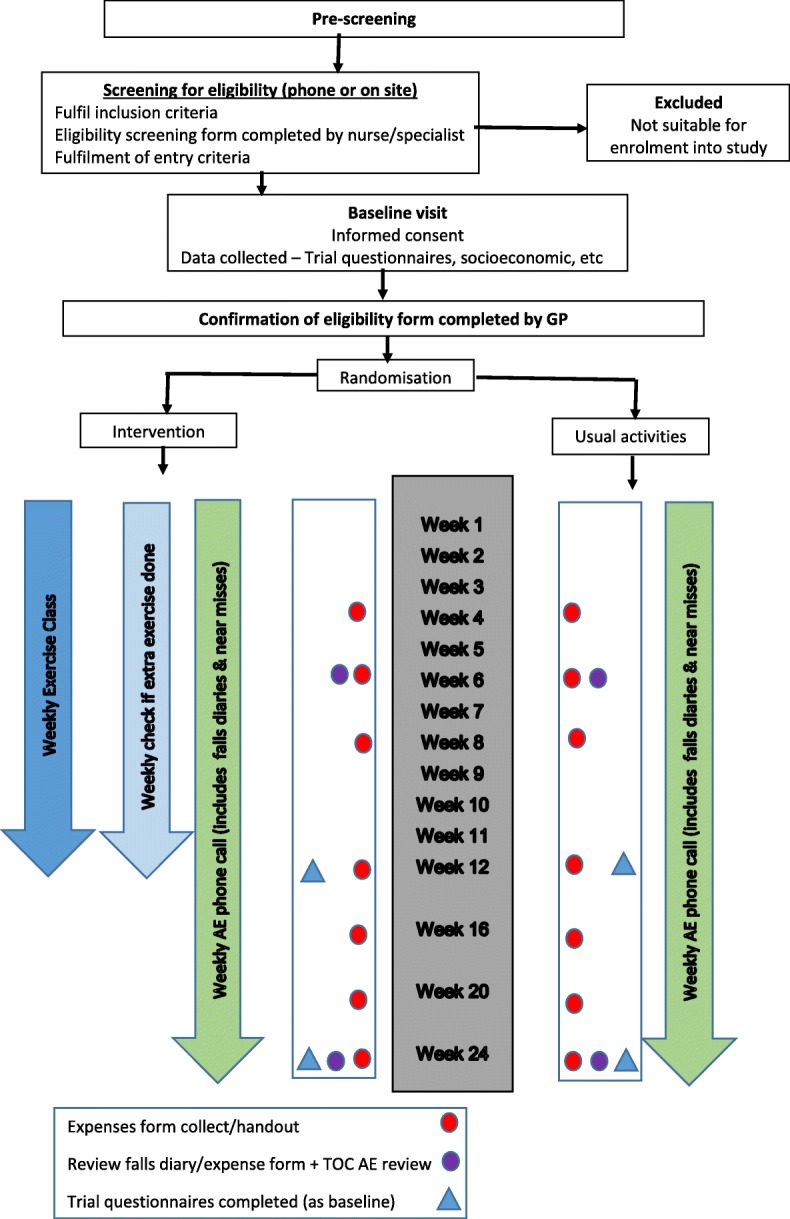
Schematic Representation of the Randomised Controlled Trial

The selected outcome measures were standardised assessment instruments that have been used in falls research. These were assessed at baseline, 12 weeks and 24 weeks (Table 1, Fig. 1). Fear of falling (FoF) was selected as the primary outcome variable. The Short Falls Efficacy Scale – International (SFES-I), captures the participants’ concerns about doing everyday activities without falling [39].

**Table 1:** Trial Procedures

	Intervention	Control
Weeks 1–12	Daily completion of falls diary 1 h weekly exercise session Advice to carry out up to two hours of additional home exercise per week Weekly telephone call to/from researcher to record any adverse events Completion of falls resource/expenses form with the researcher (if required).	Daily completion of falls diary Weekly telephone call to/from researcher to record any adverse events. Completion of falls resource/expenses form with the researcher (if required).
Week 12 ((+/− 2 weeks)	Following information collected: Co-morbidities, current medication, any changes in socioeconomic information, and incidental costs (intervention group only) and the outcome measures were completed.	Information collected as per intervention group
Weeks 12–24	Daily completion of falls diary Weekly telephone call to/from researcher to record any adverse events. Completion of falls resource/expenses forms with researcher if required	Information collected as per intervention group.
Week 24 (+/− 2 weeks)	Assessed on all measures	Assessed on all measures

Secondary outcome measures included: Activity avoidance [40]; Timed Up & Go test (TUG), [41] Falls Risk (FRAT) [42]; Physical Activity (Phone-FITT) [43]; Loneliness (Six-Item Scale for Overall, Emotional, and Social Loneliness) [44]; Hospital Anxiety and Depression Scale (14 item) [45]; Work and Social Adjustment Scale (WSAS) [46]; Health related quality of life (EQ-5D-5 L) [47] and quality of life (ICECAP-O) [48]. Currently performed physical activities/exercise were also assessed using a short bespoke self-report inventory. Because of their visual impairment, assistance was often required to fill in the questionnaires, either by the researcher reading out the questions and filling out the forms, or by providing the questionnaires in a format accessible to the participant. Where no more than 20% of questions were missing or uninterpretable on specific scales, the score was calculated by using the mean or median value (as appropriate) of the respondent-specific completed responses on the rest of the scale to replace the missing items [49].

Each participant completed a falls diary each week, with assistance from a Researcher, during a weekly telephone call. Details of any adverse events and near misses were also recorded at this time and are described in the safety analysis. When a fall did occur, the researcher completed the falls resource/expenses form on behalf of the participant retrospectively during the weekly telephone at 12 and 24 weeks follow up.

### Sample size

As this was a feasibility study, a formal power calculation was not appropriate. We aimed to obtain a minimum of 30 responses in each trial arm at 6 month follow-up to estimate critical parameters to the necessary degree of precision [50]. To provide feasibility data, we aimed to recruit a total of 80 community-living VIOP to allow for loss to follow-up.

### Adherence

Adherence to the group exercise programme (register) and home exercise programme (self-report) was assessed. Participants were classed as adherent to group exercise if they attended nine out of 12 of the group sessions [36].

### Economic evaluation

Cost effectiveness analysis was rehearsed from an NHS and personal social services perspective using the EQ-5D-5 L and ICECAP-O as outcome measures and via a health economic self-report service receipt inventory. Costs of the intervention were micro-costed using the Violet feasibility study records. For each trial participant, all components of treatment costs stratified by category of resource use were computed by multiplying units of resource use by their unit costs. These were then summed over all resource use categories to obtain a total cost for each participant. This was then used to generate the average cost per patient in each arm of the trial. All unit costs were expressed in GBP (£) and valued at 2015–16 prices. The mean cost of the intervention across the two sites was estimated. Utilities were estimated and reported for each trial arm using means, standard deviations, the median and the range.

### Fidelity of the intervention

Instructors submitted basic lesson plans for the 12-week programme prior to the start of the intervention. Their adherence with the course content (fidelity) was assessed by a researcher (SG) attending 20% sample of exercise sessions. A standardised checklist was used, similar to that used in a previously published trial [35], and these sessions were videotaped for quality assurance purposes.

### Safety

All adverse events judged as having a reasonable suspected causal relationship to a study procedure (i.e. definitely, probably or possibly related) were considered to be related adverse events. The opinion of a physician was sought if required. Severity of all AEs was graded on a three-point scale of intensity (mild, moderate, and severe). Expected adverse events included: Fall/trip/slip and its consequences: cuts and abrasions, soft tissue injury, fracture; muscular/joint pain associated with the above or with increased physical activity; minor illness not requiring GP intervention (cold, flu etc.); minor illness requiring GP intervention (chest infection, urinary tract infection etc.)

Any serious adverse events were recorded until a participant reached their 24-week follow-up visit and included injurious falls, serious falls and/or hospitalisation due to falls.

### Statistical analysis

Since this was a feasibility trial, the main analyses were descriptive, in order to inform the design, choice of primary outcome, sample size and approach to analysis for a future definitive study. The main outcomes were feasibility outcomes; the numbers of eligible participants seen over the recruitment period, and the resulting rates of recruitment, compliance with randomisation, and data completion were presented. Data completeness of the instruments and any potential bias in the completion of follow-up data to inform the choice of instruments in a future trial was ascertained. The majority of the outcome data is presented in simple descriptive tables, presenting percentages, means and standard deviations or 5-number summary (as appropriate), for each arm of the study.

There was potential for clustering effects, particularly class-based clustering in the intervention group and this study aimed to investigate and estimate the size of any such effects.

## Results

### Identification of potential participants

In Newcastle, a dedicated member of NSBP telephoned members aged over 60 years to gain permission to forward their contact details to the research team. It is not known precisely how many potential participants were approached by NSBP, but anecdotal information suggests that over two hundred calls were made. From these, thirty-five expressions of interest were passed to the researchers. The primary source of potential participants at the Glasgow site was Visibility. Anecdotally, over one hundred direct contacts were made by Visibility staff, and the research team received forty-eight expressions of interest. The process of identifying potential participants was time consuming and incumbent upon the resources of the third sector organisations.

Identification of participants from the RVI Low Vision Clinic in Newcastle initially proved problematic: very few of the staff referred potential participants to the researchers, only seven were initially identified. After the involvement of ECLOs, fifteen further potential participants were identified, and their expressions of interest were forwarded to the research team. No screening or recruitment took place from the Low Vision Clinic in Glasgow as it was closed over the period of recruitment.

All participants who had expressed an interest in the pilot trial were contacted by the research team and assessed for eligibility in a consistent manner.

### Participant flow

**Fig. 2:**
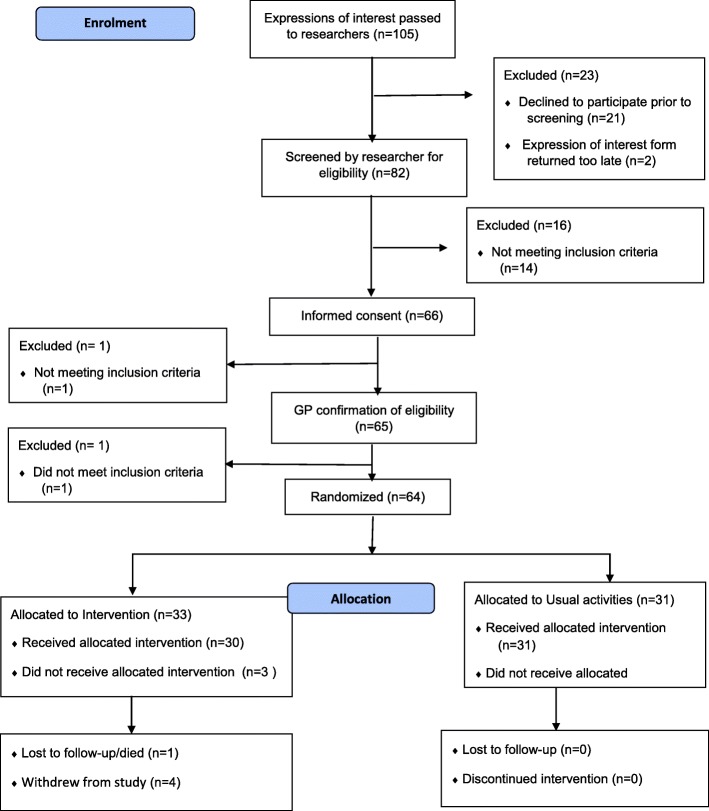
CONSORT Diagram of the VIOLET Study

Participant flow is illustrated in the CONSORT diagram in Fig. 2.

After assessment for eligibility, 68 people were asked if they would be willing to be randomised and 66 agreed to do so (97%). This consent rate was much higher than the target of 50%, but will be biased upwards as a number implicitly declined to enter trial at an earlier stage when they failed to send back an expression of interest.

Recruitment to the trial took place between June and November 2015. Recruitment was closed when 64 participants had been randomised, which was below the target of 80.

After randomisation, 33 VIOP were allocated to the intervention arm, and 31 to usual activities. Of the 33 VIOP allocated to the intervention arm, 3 did not attend any classes: 2 of those who didn’t attend any classes nevertheless provided study data. During the study, 1 person was lost to follow-up and 4 people in the intervention arm withdrew completely from the study. The remaining 59 subjects provided data that was included in the statistical analysis: this was only slightly below the target of follow-up data on 60 participants.

### Baseline participant characteristics

Demographic and baseline characteristics at randomisation were compared across treatment groups (Tables 2 and 3). The distributions of demographic variables were similar across the trial arms: the only noticeable difference was that there were more who lived alone in the usual activities arm.

**Table 2:** Baseline demographic characteristics, by treatment arm

Variable	Intervention Arm (*n* = 33)	Usual activities Arm (*n* = 31)
Gender		
Male	14 (42%)	11 (35%)
Female	19 (58%)	20 (65%)
Age (years)		
Median (IQR)	80 (75, 87)	78 (68, 83)
Mean (SD)	79.3 (8.7)	76.5 (9.7)
Range	61–95	62–95
Participants’ first language:		
English	32 (97%)	28 (90%)
Other	1 (3%)	3 (10%)
Ethnicity		
White	33 (100%)	29 (94%)
Asian or Asian British	0 (0%)	2 (6%)
Marital status:		
Married/living as married	16 (48%)	8 (26%)
Living with other family members	0 (0%)	2 (6%)
Living alone	11 (33%)	16 (52%)
Widowed	6 (18%)	5 (16%)
Employment status		
Full time employment	0 (0%)	1 (3%)
Retired	29 (88%)	28 (90%)
Other	4 (12%)	2 (6%)

**Table 3:** Baseline numbers of self-reported co-morbidities, by treatment arm

Comorbidities		Intervention Arm (*N* = 33)	Usual activities Arm (*N* = 31)	Total (*N* = 64)
Any comorbidities reported	Yes	25 (76%)	20 (65%)	45 (70%)
	No	8 (24%)	11 (35%)	19 (30%)
Numbers of comorbidities per participant	Min	0	0	0
	LQ	1	0	0
	Median	2	2	2
	UQ	5	6	5
	Max	9	13	13

### Delivery of intervention and intervention adherence

A staggered start was used to facilitate the running of the first set of classes in both Newcastle and Glasgow. This enabled those for whom the GP confirmation of eligibility had not been returned promptly to start at any time within the first three weeks and continue to complete the twelve sessions. In Newcastle, a third set of classes was provided for those who had been recruited later, or whose eligibility checks took a long time to complete. In Glasgow, the class sizes were six and nine respectively and, in Newcastle, class sizes were six, five and four respectively.

Four withdrew completely from the study and attended 0, 2, 3 and 11 classes prior to withdrawing. One participant died while in the trial, after attending 6 exercise classes. One participant withdrew from the intervention after 3 classes but continued to provide trial data. Two additional participants randomised to the intervention arm did not attend any classes as their GP consent was not received in time, but continued to provide follow-up. Despite this, 76% attended 9 or more classes, which was one of the feasibility criteria for a future trial.

Table 4 summarises the number of sessions attended by participants randomised to the intervention arm on the basis of class registers, and how often they exercised at home (self-report) with its frequency and duration. It can be seen that on average they spent 50 min per week, though there was a large variation in the amount of time spent exercising. This was much less than the 2 h per week that they were encouraged to so.

**Table 4:** Summary statistics for the exercise classes attended and home exercise

	min	LQ	Median	UQ	max
Number classes attended	0	9	10	12	12
Weeks when exercised at home during intervention^a^	0	5	9	11	14
Average weekly exercise frequency^b^	0	1.8	3.4	4.6	6.7
Average exercise duration per week (min)^b^	0	17.3	50.4	75.7	122

^a^ ‘during intervention’ is any calls made in the period between 1st exercise class and last class + 7 days

^b^ is the average frequency or duration for all weeks when participant reported exercising at home in the valid timeframe (note that these include zeros for weeks where no exercise at home was done)

### Data completeness

Sixty out of 64 (94%) provided data at 12-week visit (four had withdrawn completely from the study at this point). Fifty nine out of 64 (92%) participants completed the 24-week visit (four had withdrawn completely and one was lost to follow up from the study at this point). In two cases, assessments were completed outside the two-week limit, due to other commitments or extenuating circumstances.

All those participants in the intervention arm remaining in the trial at each time point completed each of the questionnaires. There were two occasions when participants in the usual activity arm only partially completed a questionnaire (but this was still usable using missing data rules), and two occasions on which whole questionnaires were not completed. The elements of the phone-FITT questionnaire were completed for all those remaining in the trial at each time point. This suggests that all the chosen scales were suitable for use in a future trial, though the need to have researchers help participants, proved time consuming.

### Questionnaire data throughout the trial

Table 5 summarises numeric outcome measures by trial arm and data collection point, for the SFES-I, WSAS, FRAT and phone-FITT. It can be seen that, based on the SFES-I score, the majority of participants had low or moderate concern over falling at baseline. The change from baseline in SFES-I at 12 and 24 weeks was minimal in both arms. There was a very wide range of WSAS impact scores at baseline, but the median changes over time were small. For FRAT, the scores were generally low at baseline and showed little change over time. Using the Phone-FITT summary, it can be seen that typical physical activity levels rose slightly over the follow-up period in the intervention arm, and less so in the control arm, though no formal comparison was made. These illustrate that concern over falling (the proposed primary outcome for a definitive trial), and assessment of falls risk was low in the participants recruited, so there was little progress to be made by any intervention.

**Table 5:** Numeric outcome measures by trial arm and data collection point (weeks 0, 12 & 24)

	Week	Intervention arm						Usual activities arm					
		n	min	LQ	Med	UQ	max	n	min	LQ	Med	UQ	max
SFES-I (concerns over falling) *Scores 7–28*: *Higher scores= > more concern*	0	32	7	8	9	10	15	31	7	7	9	11	23
	12	29	7	8	9	11	14	30	7	7	8	10	20
	24	28	7	7	8	10	21	31	7	7	8	11	17
Impact of visual impairment (WSAS) *Scoring (0–40):* *Higher scores = > more impact*	0	32	0	5.6	11.9	18.1	27.5	30	0	5.0	12.5	23.8	38.8
	12	29	0	10	15	20	35	31	0	3.8	11.3	17.5	37.5
	24	28	0	12.5	20	25.6	40	31	0	5	15	26.3	33.8
Falls risk assessment tool (FRAT) Scoring (0–5): *Higher scores = > more risk*	0	33	0	0	1	3	5	31	0	1	1	3	4
	12	29	0	1	1	3	4	31	0	1	2	3	4
	24	28	0	1	1	3	4	31	0	1	2	3	4
Phone-FITT Total Frequency and Duration (FD)	0	33	0	34	49	89.5	296	31	1	16	42.5	66	222
	12	29	0	41	55	96	209	31	0	25	47	67	377
	24	28	9	30.8	52.1	100.5	410	31	0	18	43	66	1506
Functional test (TUG) Time in seconds to complete the test	0	33	8.8	9.8	13.3	16.8	35.6	31	7.4	10.7	13.3	20	120
	12	28	8.9	11	13.5	18.4	30.2	31	6	11.1	17	19.2	975
	24	28	8.2	10.3	13.8	16.3	28.5	31	6.6	10.4	15.1	18.2	100

### Safety analysis

A total of 180 Adverse Events (AEs) were reported; these were categorised as 16 Serious Adverse Events (SAEs) and 164 AEs. The majority of AEs reported were due to minor illness and unrelated to the intervention.

There were 81 reported AEs in the intervention arm and 83 in the usual activities arm. No AEs or SAEs occurred during the exercise classes. There were 9 SAEs in the intervention arm and 7 SAEs in the usual activities arm. Of the 9 SAEs in the intervention arm, 4 were fractures due to falls. There were no fractures due to falls in the usual activities arm. However, of the 4 participants who sustained fractures due to falls in the intervention arm, 2 of these participants did not actually begin the intervention and had a self- reported history of repeated previous falls. With regard to the other two participants in the intervention arm who sustained fractures: one had taken part in 7 exercise classes when they tripped and fell in their own home, whilst the other had taken part in 10 exercise classes when they tripped and fell outside. There appears to be no evidence of a link between taking part in the intervention and being at greater risk of a fracture due to a fall. One intervention participant died during the study, but the death was unrelated to the intervention. This highlights that these VIOP had multiple medical conditions and syndromes of ageing that affected their participation.

### Economic evaluation

Data regarding the intervention itself and the associated costs were fully recorded. Though 31 falls were reported (in 20 participants), only six participants utilised health and social care interventions post-fall, with none of these being quantifiable in terms of costs, due to missing data regarding the type of intervention utilised.

The average total cost of the intervention per patient across both sites was £310 and is shown in Table 6.

**Table 6:** Costing of intervention

Resource use	Cost (£)	Unit	Source
Costs of Consumables			
Yoga Mats and exercise bands	420	Total cost	VIOLET study files
DVDs and CDs	65	Total cost	VIOLET study files
Costs of Staff Time			
Newcastle PSI (12 sessions × 3 cycles)	66.66	Per hour	HealthWorks
Glasgow PSI (12 sessions X 2 cycles)	61.50	Per hour	LLT
Glasgow PSI set up time (0.5 h per session for 24 sessions)	30.75	Per 30 mins	LLT
Glasgow PSI travel and parking costs (per session)	7.24	Per session	LLT
Costs of Room Hire and Refreshments			
Newcastle (3 × 12 week cycles)	1925	Total Cost	HealthWorks
Glasgow (2 × 12 week cycles)	1476	Total Cost	Visibility

KEY: *PSI* postural stability instructor, *LLT* Later Life Training

Table 7 shows the EQ-5D-5 L utilities and ICECAP-O capability scores for both trial arms at baseline and each of the two follow up periods. Results show that for both treatment groups at baseline, EQ-5D scores were poor, indicating ‘a state worse than death’ with the health state being worse in the intervention group.

**Table 7:** EQ-5D-5 L utility scores and ICECAP-O for health-related quality of life by trial arm

	Intervention Arm				Usual Activities Arm			
	N	Mean (SD)	Median (IQR)	Range	N	Mean (SD)	Median (IQR)	Range
EQ-5D-5 L Utility Scores								
Baseline	28	−0.23 (0.25)	−0.25 (0.39)	0.83	31	−0.15 (0.24)	−0.19 (0.18)	1.15
12 weeks	28	−0.2 (0.22)	−0.2 (0.29)	0.78	31	−0.12 (0.27)	−0.15 (0.35)	0.95
24 weeks	28	−0.21 (0.27)	−0.26 (0.47)	0.9	31	−0.06 (0.28)	−0.09 (0.43)	1.14
ICECAP-O Capability Scores								
Baseline	27	0.79 (0.14)	0.84 (0.22)	0.46	31	0.77 (0.12)	0.77 (0.2)	0.43
12 weeks	27	0.8 (0.11)	0.82 (0.13)	0.46	31	0.8 (0.13)	0.83 (0.19)	0.48
24 weeks	27	0.8 (0.14)	0.83 (0.14)	0.67	31	0.78 (0.15)	0.82 (0.24)	0.54

Compared to baseline the utility, scores at both follow up periods were improved for both groups but still showing average health related quality of life scores being poor. The range of utility scores were much larger in the usual activities arm compared to the intervention arm with more observations showing positive health related quality of life.

The capability score in the intervention arm was slightly higher compared to the usual activities arm at baseline. At 12 weeks, capability was slightly higher with both groups having on average 80% capability.

At 24 weeks, capability was maintained at an average of 80% capability in the intervention arm, with a slight reduction in the usual activities arm (78% capability).

Table 8 identifies the main issues raised and implications for a future trial.

**Table 8:** Summary of issues raised and implications for a future definitive trial

Issues	Findings	Implications for future trial
Identifying potential participants	a) Via third sector organisations Not clear how many had been contacted Very time consuming Impractical to use research staff b) Via low vision clinic Only few expressed interest when Eye Clinic Liaison Officers involved	Would need to support third sector staff more Debatable whether Eye Clinic is worthwhile route First contact may be better via GP
Did eligible participants consent?	66/68 (97%) of those eligible consented to enter trial: exceeded target of 50% Estimate biased upwards since some declined before being screened	Unreliable estimate from this pilot trial
Met recruitment target?	Randomised 64 participants to trial: below target of 80	Recruitment was more difficult than anticipated: need to improve procedures
Compliance with intervention	25/33 (76%) completed at least 9/12 group sessions: exceeded target of 70%	Compliance with exercise class regime is possible
Home exercise duration per week	Median duration 50 min: encouraged to exercise for 120 min	Need to find ways of encouraging participants to exercise at home for longer
Intervention	FaME intervention successfully adapted for VIOP	Need exercises appropriate to ability of individual, providing sufficient challenge
Retention throughout study	59 participants retained to end of study: narrowly missed target of 60	Retention was better than anticipated
Outcome assessments completed	60/64 (94%) provided data at 12 week visit 59/64 (92%) provided data at 24 week visit: exceeded target of 70% Very few items of missing data on any scale	Once recruited, retention and data collection was very good. No problem with completion if researchers can help, but this is time-consuming
Suitability of candidate outcome measures	Suitable, howeverelements of standard assessments were occasionally inappropriate for VIOP	Other outcome assessments may additionally be included.
Safety issues?	16 serious adverse events, but none deemed due to intervention: less than target of < 10%	No safety issues
Sample size calculation for definitive trial	Calculation very imprecise because of lack of estimates of parameters for SFES-1	Better estimates necessary
Data collection for health economic analysis	Data often missing from resources form	Data collection form needs to be more structured

## Discussion

The study has shown that it is feasible to conduct a RCT of a modified FaME exercise intervention in visually impaired older people. Recruitment of eligible participants to the randomised feasibility study exceeded the pre-planned progression criteria, as did the adherence of participants to the intervention and data collection from the participants. In addition, as planned, less than 10% of serious adverse events were due to the intervention itself.

### Improving recruitment

Variation in the way participants were identified across the two study sites led to some discrepancies in the recording of eligibility for the study. We are aware that over 300 direct contacts (phone or letter) were made to potential participants, which converted to 105 expressions of interest. An accurate count of contacts would be required for future studies.

Overall, recruiting from third-sector (voluntary and community)organisations was successful, though organisational feedback suggested that the conversion rate from initial contact to expression of interest was poor. Also, one of these organisations was able to provide a dedicated member of staff for recruitment, but the other was not. Thus, identification of potential participants was more burdensome to the voluntary and community organisations than expected. In future multi-site studies, a recruitment strategy should be discussed and agreed across recruiting organisations with provision of additional resource and support. Further stakeholder involvement may improve recruitment and should be considered for a future definitive study.

It proved difficult to recruit from the NHS Low Vision clinic. Since this was a regional centre, many patients lived too far away from the exercise classes provided. On the other hand, the NHS ECLOs dedicated a member of staff to identify potential participants and pass on expressions of interest. This recruitment strategy met the progression criteria with near to 50% conversion rate, though low overall numbers. Only two of those originally found eligible for the study declined to take part.

Given the difficulties experienced in recruiting via third-sector organisations and an NHS low vision clinic, in a future study it may be advisable to use the primary care setting with the dual purpose of GP-led assessment of health exclusions and recruitment. Previous research suggests that a ‘personal’ invite by a health care professional increases uptake of community exercise classes [51].

### Delivery of the intervention

A key component of the VIOLET study was the successful delivery of the adapted FaME programme in which instructors adapted their delivery style to the needs of participants.

For each cohort, quality assurance checks were performed, ensuring fidelity of the programmes at both sites. We recommend that for future studies, participating instructors should have an opportunity to attend two workshops, to share and discuss findings regarding successful delivery of the intervention.

Within the exercise classes, a wide range of participant ability led to some participants reporting not being physically challenged nor understanding the relevance of specific exercises. There is a need for falls prevention programmes to emphasise facilitating independence as well as other positive benefits, such as socialising and receiving useful health education, in addition to an exercise component. However, it is possible that the dose and duration of exercise classes were insufficient for many of these participants to notice tangible benefits, as many exhibited low FoF and low to moderate falls risk scores.

Guidelines recommend at least 36 h of exercise per falls prevention exercise programme, over the 12 weeks of participation, equating to a total of three hours per week [2, 29, 38]. However, VIOLET study participants spent an average of 50 min on home exercise, which only provided 1 h 50 min in total when the weekly group exercise intervention was included. Home exercising was hard to sustain, particularly once the 12 weekly group classes were completed: this concords with other research [32, 33, 35]. The VIP and VIP2UK studies [32, 33] and ProAct65+ [35], reported poor adherence to the home exercise component, although the studies suggested that stricter adherence was associated with fewer falls. Access to home exercise information could be enhanced technologically by providing audio material that can be easily paused, revisited and generally modified to individual preference, such as DAISY (Digital Accessible Information System), screen reader, voice synthesiser, MP3, talking book and Braille. Continued development of strategies to increase adherence to home exercise, or an alternative may be to offer more group sessions in a week, is recommended, rather than reliance upon home exercise. Classes may also provide a platform for an informal sharing and exchanging of broad health information. Because reduction in falls risk does require sustained engagement with exercise to maintain strength and balance as we age, irrespective of VIOP, it is important that future studies attempt to incorporate strategies to encourage behaviour change beyond the intervention. Many falls interventions attempt to do this by encouraging self management and self-efficacy with engagement in home exercise, but as engagement with home exercise in VIOP appears so challenging, other strategies may need to be adopted.

### Methodological issues

The completion rates of the outcome measures were very high. Ninety-four percent of trial participants provided data at 12 weeks and 92% at 24 weeks, although as researchers often were needed to aid completion, the time allocated to this task requires consideration. In general, these participants took longer to perform the TUG time/functional ability assessment than that reported for healthy community dwelling subjects 65–84 years [52]. Additional outcome measures, such as frailty, may be included in future, in order to indicate whether exercise is maintaining the level of resilience, even if it does not lead to an improvement. This may be more appropriate and realistic for an older population with many co-morbidities. Further, a longer follow-up period of 12 to 18 months would also be recommended in future studies to explore effects of discontinuation and longer term effects (30).

Within the VIOLET feasibility study there was no assessment of visual impairment. This was largely a pragmatic decision; however, this is recommended for future studies as it would allow an assessment of whether a VIOP might be able to join a mainstream class, or whether they might require one to one intervention, or more intensive supervision. The inclusion criteria did not allow screening out for ‘deafness’. People who are profoundly deaf and have a VI are difficult to accommodate in a group setting. The assessment of whether potential participants were ‘physically able to take part in a group exercise class’ category was, on the whole, carried out when face to face, and thus open to individual interpretation. This was also the case when assessing the participant’s ability to walk indoors and outdoors with or without aid. Mixing together able and less able participants, impacts on the challenge and potential effectiveness of the programme. Stratification by functional ability, as well as falls risk, is recommended for a definitive trial.

There were a number of potential participants who ‘self-reported’ an uncontrolled medical condition as reason for exclusion, however the degree of concordance with a GP was not assessed. If recruitment were to be carried out in primary care in a future study, the assessment would be carried out by a GP, rather than by a participant or a researcher, who may not be medically trained, although our method replicates current practice in falls services.

Although the progression criteria to judge the feasibility of progressing to a full trial were all met, suggesting that this intervention could be taken to a full study, recommendations from the research team and participants suggested that most VIOP in the pilot trial could have been integrated into a mainstream class. Training (CPD) of instructors to accommodate a range of VIOP in their mainstream sessions is minimal and easy to facilitate through online training so would be affordable and have sustainable reach. Only those with multiple co-morbidities (such as extreme deafness or extreme frailty) required significantly more supervision. Mainstream classes can be much larger, so if VIOPs were to join, extra supervisors may be necessary.

### Economic evaluation

The current feasibility study has shown that whilst it is possible to collect most of the data necessary for a full cost-effectiveness analysis of the exercise intervention compared with usual care (cost of intervention per se, utility values and capability values), there were some practical issues in accessing information regarding participant self-reporting of resource use post intervention. It is unclear whether this was due to their visual impairment. Participant inputs on use of health and social care services and broader service use were often missing, despite telephone calls by researchers. The data collection instrument for this was the participant self- reported Falls Resources / Expenses Form (including informal care givers time). This was based on a series of open ended questions as it was initially thought by study team that carers filling out the form would find these types of questions easier to respond to. However, from this data capture form, it was unclear whether an absence of recorded data signified missing or whether participants had not actually received any formal care. Furthermore, detail of the type of care was also lacking, rendering any estimation of costs of the use of health and personal social care resources difficult. The use of a more structured previously piloted data collection tool may have mitigated against some of these issues though it is well documented that reliance on patients/carers as a data collection method is limited by biases in recall, nonresponse, and evasiveness [53]. Missing data is a common problem for economic evaluations that run alongside clinical trials [54].

The HRQoL scores showed that participants in both arms of the trial at all time points were poor. However, capability scores were towards the high end of being capable. The ICECAP measures potentially offer a broader assessment of quality of life and well-being, in comparison to measures routinely used in economic evaluation, such as the EQ-5D-3 L [55]. This broader assessment may allow measurement of the full effects of an intervention or treatment. Previous research has indicated that the ICECAP-O (for older people) and EQ-5D-3 L measures provide complementary information and are not substitutes [56]. The same may be true for the EQ-5D-5 L however, this is currently not addressed in the literature.

### Limitations

The main limitations were that the sample recruited typically exhibited low to moderate falls risk and relatively low fear of falling. The intervention may have benefited higher risk fallers and these should be targeted for recruitment in future studies. Recruitment proved difficult and we did not recruit the originally planned sample of 80 participants, though retention was better than anticipated.

It is possible that there was potential bias by having the weekly telephone call, which may have acted like an intervention in itself. There was no assessment of visual impairment and we only recruited English speaking participants. These findings limit the generalisability of the findings to a wider, more ethnically diverse population. There was a lack of appropriate estimates upon which to calculate sample size for a definitive trial.

## Conclusions

It was possible to adapt successfully an existing, widely used exercise intervention for falls prevention (FaME) for people with visual impairment. Adherence to the intervention was high with very low attrition rates. It was to be expected that there would be no difference between the two groups in the main outcome measure, fear of falling, but a further two reasons may have contributed to the finding: there was possible a sub-optimal dose of exercise and the majority of participants were found to be of low risk. Although the progression criteria were met, a future definitive trial should consider the development of strategies to increase physical activity and structured exercise at home, in order to reach recommended levels of activity. Stratification of those with low, moderate and high falls risk should also be explored and that should include an assessment of visual impairment and functional ability as those with better functional vision may be able to be integrated into mainstream programmes. A recruitment strategy should be discussed and agreed across recruiting organisations which should also could include primary care practices and involve the multidisciplinary team.

## Abbreviations

AE: Adverse Event
DAISY: Digital Accessible Information System
ECLO: Eye Clinic Liaison Officer
FaME: Falls Management Exercise
FoF: Fear of Falling
FRAT: Falls Risk Assessment Tool
GCP: Good Clinical Practice
GP: General Practitioner
HADS: Hospital Anxiety and Depression Scale
ICC: Intra-class correlation coefficient
ICE-CAP-O: ICEpop Capability Measure for Older People
NHS: National Health Service
NIHR: National Institute of Health Research
NRES: National Research Ethics Service
NSBP: Newcastle Society for Blind People
NuTH: The Newcastle upon Tyne Hospitals NHS Foundation Trust
PAG: Project Advisory Group
PHR: Public Health Research
PI: Principal Investigator
PIS: Patient Information Sheet
PSI: Postural Stability Instructor
QoL: Quality of Life
R&D: Research and Development
RCT: Randomised Controlled Trial
REC: Research Ethics Committee
SAE: Serious Adverse Event
SFES-I: Short Falls Efficacy Scale - International
TMG: Trial Management Group
TOC: Trial Oversight Committee
TUG: Timed Up and Go
VI: Visual Impairment
VIOLET: Visually Impaired Older Adults Exercise Programme for Falls Prevention
VIOP: Visually Impaired Older Person
WSAS: Work and Social Adjustment Scale
